# Selected Psychological Aspects of Meat Consumption—A Short Review

**DOI:** 10.3390/nu10091301

**Published:** 2018-09-14

**Authors:** Klaudia Modlinska, Wojciech Pisula

**Affiliations:** Institute of Psychology, Polish Academy of Sciences, 1 Jaracza St., 00-378 Warsaw, Poland; wojciech.pisula@wp.pl

**Keywords:** meat, protein, amino acids, phytoestrogens, digit ratio, autism, food neophobia

## Abstract

Eating meat is deeply entrenched in Western culture. It is often associated with wealth and a highly nutritional diet; and for many people it is also an established habit that is difficult to change. The second half of the 20th century was a period of rapid growth in meat consumption, which resulted in intensified meat production. At the same time, eating meat has recently become subject to criticism for health-related, environmental or humanitarian reasons. This review aims to signal the potential consequences of a change of diet or switching to diets that are rich/poor in certain ingredients on the functioning of the hormonal and nervous system, which translates into changes in mood and behavior. This paper discusses the psychological phenomena which underlie the difficulty of changing one’s food preferences and problems encountered while adding new products to the daily diet. Finally, this study summarizes the limitations of modifying eating habits that have resulted from established attitudes and habits.

## 1. Introduction

Since prehistoric times, humans have based their diet on the meat of animals that inhabited their environment [1]. There are grounds for assuming that the willingness to eat meat was a significant factor shaping the final stages of human evolution [2,3]. The 20th century saw a particularly pronounced increase in meat consumption, thanks to economic growth, developments in meat production technology, and intensified urbanization. A meat-based diet became the symbol of wealth, and meat consumption was regarded to be the best way to satisfy one’s nutritional needs [4]. On a global scale, the fast and constant growth of the human population results in the need to increase food production. However, the intensive mass-scale animal-based production is linked to numerous strains on the environment. Intensive meat production also requires new areas and resources (e.g., huge amounts of water, electricity, fuels of transport, etc.). To reduce the demand for additional land for agriculture, production technologies used nowadays involve genetic modifications and the use of chemical substances (e.g., pesticides, antibiotics, hormones). In 2013, FAO (The Food and Agriculture Organization) reported that livestock farming accounted for 14.5% of anthropogenic greenhouse gas emissions [5]. It seems justified to say that intensive animal-based production in response to the growing demand for meat and animal-based products contributes to the devastation of the natural environment and plays a significant part in climatic change.

In light of these issues, it seems necessary to decrease meat consumption and promote a massive change of eating habits in favor of a sustainable diet. It must be borne in mind, however, that a meat-based diet not only influences the health and nutritional level of individuals, but may also shape the communities’ psycho-social profiles [6]. In addition, reducing meat consumption may not be achieved exclusively by eliminating animal-based products from the diet without introducing a well-balanced and nutritionally adequate diet (e.g., plant-based substitutes) and without close monitoring of how plant-based diets are supplemented with certain ingredients found primarily in meat (e.g., vitamins B12 [7]). A deficiency in ingredients found in meat-based products may also affect the individual’s psychological state and behavior [8]. On the other hand, consuming too much processed meat may have a negative impact on the development and functioning of the human body [9]. For example, the most recent study shows that consumption of nitrated dry cured meat was associated with current mood disorders (i.e., mania), and that feeding rats with meat containing added nitrate resulted in hyperactivity reminiscent of human mania, and alterations in brain pathways that have been involved in human bipolar disorder [10].

The problem of reducing meat consumption is not limited to the issue of following a balanced diet and supplementing potential deficiencies of essential elements. Eating meat is deeply entrenched in Western culture; for many, it is synonymous with wealth and a wholesome diet. It is also an established habit that is difficult to change. In addition, many people display a highly positive attitude to eating meat—they enjoy and derive pleasure from consuming it [11]. Information campaigns aimed at raising awareness of the negative impacts of meat consumption on the environment, animal welfare and human health trigger psychological defense mechanisms and fail to reduce meat consumption levels in the general population. This happens despite the fact that switching to a vegetarian diet is now commonly regarded as a highly commendable action, which speaks well of the person who has decided to do so [12].

## 2. Method

The purpose of this paper is to provide an overview of selected psychological problems associated with introducing changes in the diet, as well as to discuss the potential influence of meat consumption on human psychological state or mood. The first part of the study presents some of the well-established notions about the co-relation between nutrition and psychological variables. There are subsequently set alongside several recent hypotheses and approaches. The second part concentrates on the main psychological constraints on implementing any changes in the diet. The psychological phenomena we describe constitute the main and most influential ideas in the field. We also summarize the conclusions presented in important reviews of previously published studies.

Given its broad thematic scope, this study takes the form of a short review. In addition to summarizing the key points of the topics discussed, our paper highlights those areas which are currently undergoing dynamic development, and points to several aspects that would benefit from further research. The short review form also allows us to suggest some perspectives that are not yet mainstream ideas in this specialized research field.

## 3. Protein

Although protein deficiency is rare in developed countries, it can occur when switching to a vegetarian diet via significant dietary restrictions [13]. In addition, the high demand for high-quality protein occurs in, e.g., athletes [14]. This problem also affects many developing countries, where a strictly vegetarian diet (often forced by economic conditions) leads to serious nutritional deficiencies and related developmental disorders [15]. The adverse effects of the protein deficit have also been confirmed by numerous studies conducted on animals. The classic study by Cowley & Griesel [16] pointed to serious emotional disorders in rats born to mothers kept on a protein-deficient diet. More recent studies also point to the impact of protein deficits on behavior. For instance, the offspring of female rats kept on a low-protein diet displayed changes in the control of appetite, distorted perception of palatability, and a preference for fatty foods [17]. Studies by Zamenhof and colleagues [18] also suggest behavioral disorders in the offspring of protein-deficient female rats. Nevertheless, there are also reports on the possible therapeutic significance of a reduced-protein diet. Experiments on dogs suggest that reducing the level of protein in the diet may be useful in dogs with territorial aggression [19]. Additionally, a case study of mentally retarded females with phenylketonuria described a significant behavioral improvement after introducing a restricted protein and high-energy diet [20]. However, it should be noted that this study presents only one specific case and may not be confirmed in other patients. In addition, animal-based food is important for growth, recovery rate and cognitive abilities in undernourished children [21]. The addition of meat to snacks for school children were also recommended as a remedy for a deficiency in micronutrients in developing countries [15]. In population studies, meat consumption in human children correlated with cognitive and academic performance [22].

A meat-based diet is linked not only to a high protein content, but also to high fat levels. For instance, a high-fat diet in female rats has been associated with alterations in maternal care and pups’ milk consumption during the early postnatal period [23]. Generally, an unbalanced diet (e.g., protein restriction, micronutrient restriction and excess fat feeding) may result in the programming of appetite in offspring and influence their susceptibility to obesity through the re-modelling of hypothalamic structures that control feeding and through programming of the expression of genes involved in responses to orexigenic hormones [24].

## 4. Amino Acids

Many of the indispensable amino acids come from one’s diet, as they are not synthesized by the human body. Some of the indispensable amino acids are closely linked to mood, since they are precursors for neurotransmitters [8]. As a result, they play a key role in brain functioning, while their deficiency may lead to pathologies, i.e., mood disorders. For instance, tryptophan is the precursor for serotonin, which plays an important role in the areas of the brain that are responsible for emotional regulation and sleep. What is more, serotonin influences appetite regulation and regulates the connection between appetite and mood. The rate at which amino acids access the neurons which synthesize serotonin has a direct impact on the rate at which they are processed into neurotransmitters. Providing amino acids directly or in meals increases the amount of tryptophan that reaches the neurons, which results in a sudden increase in serotonin production. Such manipulations lead to observed changes in the brain function, and, consequently, in behavior. For example, the administration of tryptophan can modify sleep and mood through the stimulation of serotonin production and release [25].

Other important amino acids include tyrosine, which is the precursor for dopamine and noradrenaline. Dopamine contributes to the regulation of motivation, concentration and the ability to experience pleasure [8]. Studies show that the ingestion of protein-containing foods increases the level of tyrosine in blood, and, as a result, the level of dopamine in the brain [26].

The intracellular deposits of free amino acids are limited and it is necessary to ingest amino acids with meals daily. Animal-based products contain all the indispensable amino acids. However, an appropriate combination of plant-based products, such as cereal grains and some vegetables (e.g., soy, beans), may, to a certain extent, substitute the animal-based amino acids in the daily diet [27]. The protein quality of a food in terms of the content of indispensable amino acids can be determined by the Protein Digestibility-Corrected Amino Acid Score (PDCAAS)—[28]. It is a measure of a protein’s ability (through digestion) to provide adequate levels of indispensable amino acids for humans [29]. Most animal proteins have a PDCAAS close to or equal to 1.0 (which is the maximum score). Similar value was calculated for soy protein. However, other plant proteins have lower PDCAAS. This kind of scoring of amino acid content in the food seems highly useful when determining the quality of protein of different origin in mix diets.

## 5. Phytoestrogens

Eliminating or greatly reducing the amount of meat in the diet makes it necessary to substitute animal protein with plant protein (e.g., soybean). However, this type of diet is particularly rich in phytoestrogens, which is not without impact on the human body (see e.g., [30,31]). Studies show that in postmenopausal women, a phytoestrogen-rich diet increases their serum levels of sex hormone-binding globulin (SHBG), which alleviates menopausal symptoms [32]; this is highly likely to influence mood and behavior. The wide spectrum of effects that plant-derived bioactive compounds have on the body, including the nervous system, is described in a study by Sirotkin & Harrath [33]. The authors refer to research which demonstrates that soy isoflavones and phytoestrogens of black cohosh, kudzu, kava, licorice, and dong quai may affect neurons via both steroid receptor and 5-hydroxytryptamine receptor or via promotion of serotonin reuptake, i.e., through both estrogenic and serotonergic activities [34]. The authors also draw conclusions about the potential suppressive influence of phytoestrogens on Parkinson’s and Alzheimer diseases [33]. In addition, research by Lephart and colleagues [35] suggests that the consumption of dietary phytoestrogens resulting in very high plasma isoflavone levels can significantly alter sexually dimorphic brain regions, anxiety, learning, and memory. Moreover, replacement of meat protein with soybean protein may have a minor effect on biologically active sex hormones [36]. While analyzing the potential impact on dietary phytohormones and their relatively high concentration in diets rich in animal products, it should also be borne in mind that infants can digest and absorb dietary phytoestrogens in active forms [37]. Although the EFSA (European Food Safety Authority) considers soy-based formula nutritionally adequate and safe as milk-based formula [38], it should be taken into consideration that in some countries infants fed soy-based formula have high circulating concentrations of isoflavones [39]. More long-term populational studies is needed to examine the role of excessive exposure to isoflavones in early childhood [40,41]. This is significant, as neonates are generally more susceptible to hormonal disruptions than adults [37].

## 6. Prenatal Development—Digit Ratio

Since diet is subject to dynamic changes resulting from an increasing availability and supply of food products, it appears to be a key environmental factor influencing human physiology, including foetal development. Although there is no evidence for a direct influence of meat and fish consumption on behavior, there are reports of the impact of certain products on ontogenesis, and, consequently, on the individual’s psychological features. It is known that countries differ in terms of the mean intake of meat and other foods [42]. Based on the profiles of 29 countries, we managed to identify three groups of countries that differ in the consumption of key food products, i.e., meat, wheat, etc. [6]. Cluster 1, which includes countries located in the Mediterranean and Black Sea region, is characterized by a plant-based diet (wheat and vegetables) and low consumption of animal source foods (all types of meat plus eggs). Cluster 3 includes mainly Scandinavian, Northern European and large non-European countries (such as Canada, Australia, and USA). The diet in these countries is based on meat (mainly beef, poultry, and fish), while the consumption of plant products (wheat and vegetables) and eggs is relatively low. Cluster 2 includes countries of Central and Western Europe. In these countries, the consumption of pork and eggs is high, and these products constitute the main components of the diet. However, overall meat consumption is significantly lower than in Cluster 3. These clusters have been examined with respect to correlation with the prevalent indicators of sex steroids levels, which influence prenatal development.

A widely used biomarker of prenatal sex steroids’ levels is the digit ratio [43,44,45,46]. The digit ratio is the relative length of the second (index) and fourth (ring) finger (2D:4D). Sexual dimorphism in the digit ratio is first observed in utero [47,48]. It is hypothesized that the genes that control urogenital development can also influence limb formation [49]. On average, men have lower digit ratios than women [50].

Studies point to numerous correlations between the 2D:4D phenotype and differences in levels of multiple sex-dependent psychological traits [46,51,52,53]. For example, individuals with a low digit ratio score high on the dominance scale [52], mainly in relation to aggressive dominance [54]. A correlation has also been confirmed between the digit ratio and reproduction, pubertal development, strength and fitness, aggression and social behavior [45,50,55,56], as well as attractiveness for the opposite sex [57]. In addition, in societies where women have lower and men higher 2D:4D than expected for their respective sex, there is a higher representation of women in parliament and greater female workforce participation [43]. This is associated with the high dominance scores in women [52]. This may suggest that high testosterone in utero is associated with the shaping of characteristics, preferences, and abilities required in traditionally masculine professions.

Our cluster analysis [6] showed that countries with predominantly plant-based diets have high digit ratio values both in males and females, i.e., a low level of prenatal testosterone and/or a high level of prenatal oestrogen. By contrast, in countries with predominantly meat-based diets, the male pattern of the digit ratio is dominant in both sexes, suggesting high prenatal testosterone and/or low prenatal oestrogen exposure in utero. The remaining countries have average parameters for the clusters in question, with sex-typical digit ratios. These findings suggest a correlation between the type of diet and digit ratio distribution in a given population. A protein-rich, meat-based diet seems to be associated with the masculine 2D:4D, while a plant-based diet seems to be associated with the feminine 2D:4D [6]. Since the digit ratio is believed to be determined in utero through the effects of prenatal sex steroids [47,48,58], we hypothesized that diet and the respective levels of these hormones may indeed be related. This study suggests that the diets involving a high or low intake of meat, in an indirect way, may shape the socio-economic profile of some populations. 

## 7. Postponed Impact of Meat Consumption—A Hypothesis

The harmful effect of red meat and processed meat on human health has been well documented. The consumption of red meat was classified in 2015 by The International Agency for Research on Cancer (IARC) as probably carcinogenic to humans (Group 2A), while the consumption of processed meat was classified as carcinogenic to humans (Group 1)—[9]. Meat processing can result in the formation of carcinogenic chemicals, such as N-nitroso-compounds (NOC)—[59] and polycyclic aromatic hydrocarbons (PAH)—[60]. What is more, NOC can be produced endogenously in the body from nitrosatable precursors [59] derived from meat and its products. Additionally, the cooking of meat can produce heterocyclic aromatic amines (HAA) and PAH.

However, it should be noted that most studies focus on the immediate impact of the consumption of meat and meat-derived products on human health and (prenatal) development processes. Since it is highly probable that the carcinogenic effect of some of the chemicals mentioned above results from genetic mutation, it cannot be ruled out that epigenetic changes may also be involved in the process. Therefore, it is conceivable that those impacts may be observed in future generations. The impact path may be as follows: a high intake of element X leads to developmental changes in offspring phenotype manifestation. The study of poultry consumption and the correlated rise in autism prevalence [61] is an example of a potential correlation between the consumption of certain meat products and individual features.

**Figure 1:**
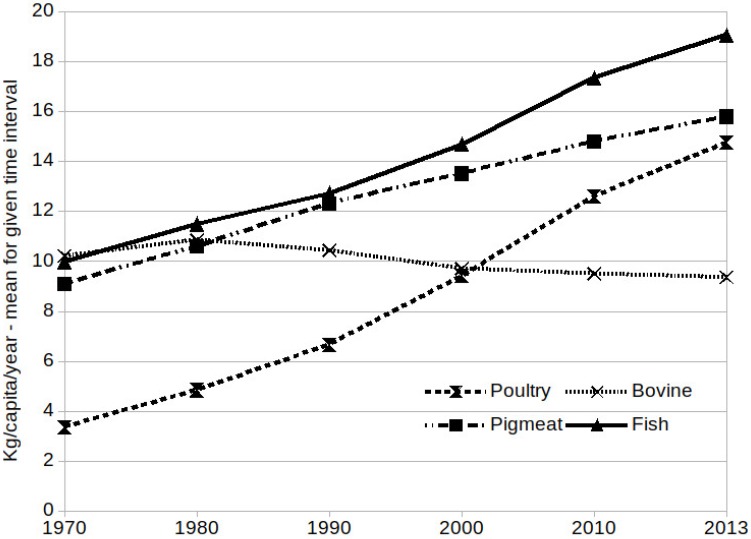
Meat consumption per inhabitant—worldwide data. Source: see [65].

Although, as in all correlational studies in general, it is hard to postulate a certain correlation between the variables studied and to draw any conclusions about causality, an interesting trend may be observed with regard to the phenomena under study. The second half of the 20th century was a period of rapid growth in meat consumption, particularly of poultry and fish (Figure 1). The increase in poultry consumption is associated with a very dynamic increase in productivity, which involved the introduction of feed additives containing ingredients stimulating muscle mass growth (e.g., hormonal elements), antibiotics to prevent infections inevitable in conditions of factory farming, and intensive genetic selection aimed at improving production value [62]. Increased poultry production correlates with an increase in autism prevalence. Although the ethology of ASDs (Autism Spectrum Disorders) is still unknown, one of the most commonly discussed hypotheses is the one proposed by Baron-Cohen ([63]; see also [64]), which points to the significant role of testosterone in shaping the child’s psychological traits. The study mentioned above [61] presents a positive correlation between the meat consumption index and ASDs prevalence. The time characteristics of the phenomena may suggest the existence of the aforementioned sequence of events: a rapid increase in meat consumption (mainly poultry) by young women (also pregnant women) and their children is associated with an increase in autism prevalence in the grandchildren’s generation, who experience a continuous significant increase in the consumption of poultry derived from intensive large-scale production.

The presented research aims to highlight the hypothesis about the possible long-term and multigenerational impact of meat and processed meat consumption on epigenetic changes manifesting in behavior. However, further longitudinal studies are needed in this regard. It seems that, in the context of the consumption of large quantities of mass-produced meat and its highly processed products, such hypotheses should be taken into account.

## 8. Food Neophobia

Food preferences are shaped already at the pre-natal stage of development [66,67], and the process continues in the weaning stage, when the child is introduced to the flavors and odors in the mother’s milk, in her breath and smell [68]. By this kind of pre-exposure, the child becomes familiar with the flavors and odors of food eaten by the mother and develops specific lifelong food preferences. The variety of flavors and odors that are available to the infant influences the child’s tolerance of new foods in later life. Unfamiliar foods are not readily accepted, and their initial intake is significantly lower than the intake of familiar foods [69,70].

The psychological barriers to consuming novel food include disgust and fear. Disgust is an emotion that elicits thoughts and behaviors that result in avoiding the objects which elicit it [71]. Things which elicit disgust differ greatly from one culture to another; they are acquired in the learning process and are rooted in social norms. Disgust correlates with the evolutionary need to avoid infection, dirt, and diseases, and may lead to fear [72]. In the case of novel foods, this fear is reinforced by the fear of the new food itself, that is, by food neophobia [73]. The reluctance to ingest novel foods is present not only in humans (e.g., [74,75]), but also in animals (see e.g., [76,77,78,79]). The need to distinguish between edible and inedible foods is of key importance to omnivore species (including humans—e.g., [80]). This problem is what Rozin [81,71] called the omnivore’s/generalist’s dilemma. In a situation where the environment offers many potential food sources, some of the food may be toxic or unpalatable. Preference for familiar foods and caution towards new foods seems to have an adaptive value.

Higher levels of food neophobia are linked to personality traits. As food neophobia correlates positively with general neophobia [75]; it is also likely that persons highly prone to the feeling of disgust will also display high levels of food neophobia, as well as a high level of general neophobia (see [82,83,84]). Persons who fit this profile are very likely to be the least willing to change their diet. In individuals with high levels of food neophobia, a forced change of diet may lead to the elimination of certain elements (e.g., animal-based products) without substituting them with new products providing similar nutrients, which may contribute to dietary deficiencies. However, products of non-animal origin trigger lower levels of food neophobia than animal-based products [85,86].

The level of food neophobia is highest in early childhood (at the age of 2–4 years) and sometimes takes the extreme form of rejecting all unknown foods and familiar foods served in an untypical manner or in a new place [87]. As a result, the introduction of a new diet in this period is extremely difficult. In addition, children with a higher level of food neophobia are particularly reluctant to eat fruit and vegetables [88]; this reluctance may persist at a later stage. Studies also show the rejection of fish by neophobic children (e.g., [89,90]).

In addition to the food neophobia, many young children have a tendency for ‘picky’/‘fussy’ eating. This consumption trend consists of limiting the repertoire of food intake to a very narrow range. This limitation applies not only to unknown foods, but also familiar and previously consumed foods. A very selective diet maintained for a long time sometimes leads to serious deficiencies and malnutrition in children [91]. 

For these reasons, a change of diet or introduction of novel foods (e.g., vegetables or fish) in children with high levels of food neophobia may be particularly difficult, and the traditional methods of influencing attitudes often prove ineffective. However, there are techniques which help reduce food neophobia in children, i.e., repeated exposure, social facilitation, peer modelling, etc., [92], but the effectiveness of these techniques is limited, and their implementation requires a lot of time and effort.

Problems with introducing a new diet apply not only to children, but also to people suffering from certain diseases (e.g., Parkinson’s Disease) and the elderly [93]. Occurring with age or during the disease, changes in the olfactory and/or taste receptors can contribute to a decline in taste pleasantness, and hence the reluctance to try new products. In addition, memory disorders that occur with age make it difficult to recognize known foods, further strengthening food neophobia and reluctance to make changes in the diet. A similar problem affects people undergoing medical treatment (e.g., during chemotherapy) or taking medicines disturbing the sense of taste.

An additional factor hindering the change in a diet is food technology neophobia. Difficult to understand for the mass consumer processes to which the food is nowadays subjected (e.g., genetic modification, nanotechnology, irradiation) may cause fear of consumption of novel products [94]. People prefer foods with a simple composition that have undergone little processing [95]. This tendency makes it difficult to introduce new products that have been developed by means of new technologies to reduce the harmfulness or limit the content of some of their components. The fear of new technologies makes people often stay with traditional food processing techniques (e.g., frying, smoking, etc.), considering them safer, as used for generations. Because of this psychological mechanism, it may be difficult to introduce novel products, which can facilitate the change of diet. On the other hand, this predisposition can be used to promote new diets by labeling them as ‘natural’, ‘traditional’, ‘simple’, etc.

## 9. Psychology of Meat Consumption

A set of personally held ideas may have an influence on decisions about the choice of food [96]. Individuals with pro-environment attitudes, concerned about the degradation of the natural world, are more likely to accept new forms of food if they are convinced of their positive environmental impact: reducing degradation and enabling a more effective use of natural resources (see [84,97]). Persons with pro-animal attitudes may take a similar stance on the subject (see [98,99]), as they pay attention to the possibility of improving animal welfare by decreasing conventional meat production.

Nevertheless, even though meat consumption has become subject to criticism in western societies due to health-related, environmental and humanitarian reasons, many people consider meat to be a desirable element of their diet and enjoy eating it. In those individuals, the reconciliation of the morally dubious aspects with their own hedonistic needs results in defence mechanisms and in rationalizing their own behavior. Rationalization allows them to maintain their own image of a decent and moral person (e.g., [100]). Joy [101] identified three main justifications that meat eaters use; they believe that eating meat is *natural, normal and necessary* (Three Ns of Justification). It is *natural*, as eating meat is a consequence of human evolution, and the human body is adapted to meat consumption. It is *normal*, as meat is a type of food commonly consumed in developed societies. It is *necessary*, as meat contains the necessary nutrients, and its consumption is indispensable for ensuring health and good physical state. These convictions are acquired in the process of socialization by means of different social media: family, religion, mass media, etc. Piazza and colleagues [11] supplemented this theory with an additional, and in their opinion, necessary N. They concluded that an equally common justification is that eating meat is *nice*. People derive pleasure from eating meat; they find it to be tasty and fulfilling.

Other responses to the current pressure to reduce meat consumption involve various mechanisms, i.e., denial (denying that animals suffer and are killed for meat); religious justification; health justification; other justifications (asserting that humans are on the very top of the food chain; that we are naturally adapted to eating meat, etc. [102]. In addition, meat eaters often avoid thinking about the suffering of animals bred for food production, and they make a mental distinction between the food and its origin—a live animal [102]. The power of this mental distinction was shown in the study by Cairns and Johnston [103]. They demonstrated that the importance of educating children about food origin stands in contradiction with the need to shield them from difficult knowledge. The awareness of animal slaughter is viewed as a threat to children’s innocence. This study indicates a problem with the introduction of general education related to diet at the school level. Although most parents are probably in favor of the education about the health aspects of the diet, it may turn out that the approach and arguments selected for this purpose will be perceived as standing in contradiction with parents’ approach to raising their children.

Studies by Piazza et al. [11] show that people who invoke the 4Ns consume more meat, are less likely to give up eating meat, and are less interested in the moral aspects of animal breeding, which makes them feel less guilty.

In addition, habit is a crucial factor which influences meat consumption levels [104]. Habitual control mechanisms pose a particularly big challenge to changing behavioral patterns [105]. What is more, a change of mind does not necessarily lead to changes in behavior. People are often unsuccessful in changing their everyday habits, including their diet. Difficulties with implementing change are not always a result of poor willpower or insufficient understanding of health issues. Failure to abandon unwanted habits may be explained by the constant availability of cues such as time of day and location, which trigger past responses [105].

High meat consumption and a positive attitude to eating meat is prevalent in most people, even though vegetarians are considered to be more virtuous, and rejecting meat is considered to be a desirable social change [12]. No significant reduction in the level of consumption of animal-based products has been generated by attempts to model this type of behavior by many well-known people or organizations, even when they act as models in different contexts. In addition, men are less likely than women to reduce meat intake or eliminate meat from their diet [12].

It should also be noted that information campaigns aimed at persuading people to reduce their meat intake and switch to a more plant-based diet yield better results among people characterized by lower emotional attachment to eating meat products. Those who are strongly attached to meat consumption display defense reactions to such practices and are less likely to change their dietary habits [4].

The attitude to eating meat is also associated with personal socio-political beliefs. The conviction that the practice of meat consumption is legitimate and just turns out to be negatively correlated with a support for animal rights and the notion that humans are animals that evolved from other animals; while the belief that animals are inferior beings and humans have the right to kill for their meat is negatively correlated with empathy [106]. What is more, sharing the above-mentioned opinions about animals is associated with ideologies unrelated to animals, such as right-wing ideologies, xenophobia, and system justification. These studies indicate that the attitude to eating meat is associated with political and social ideology, which may be an additional factor hindering the introduction of dietary changes [107].

De Boer and colleagues [108] analyzed strategies aimed at reducing the consumption of meat such as change in meat-eating frequencies (involving meat replacers) and reduction in meat portion sizes. These strategies appeal to partly different segments of consumers. Those willing to reduce the frequency of meat consumption have already eaten relatively less meat meals, used meat replacers relatively frequently, and also demonstrated a preference for plant-based proteins. Whereas consumers that prefer the reduction of meat portions strategy were more inclined to buying organic or free-range meat and provide a variety of protein sources in their meals. This study shows that changes in diet strategies must be tailored to consumers with different dietary habits. However, the availability of products that can replace meat-based meals is equally important.

## 10. Conclusions

A subject that requires research is the potential impact of fish consumption on behavior. The inclusion of fish in the diet or specific attitudes to fish are additional issues that have not received much attention. It is known that some people who decide to stop eating meat still consume certain amounts of fish (e.g., [109]). However, there is no data on the motivation to implement such changes or on the durability of such motivation. It may be assumed that the explanation may partially lie in the different ethical status of fish and farm animals.

Additionally, little is known about the long-term impact of diet manipulation on people. Substantial changes in the diet may affect not only the health status or the psycho-social profile of the population, but also future generations (e.g., in the form of triggered epigenetic changes). It should also be taken into account that recommending limiting or enriching the diet with certain ingredients cannot take place in isolation from the typical diet in a given population and without distinction between groups with different nutritional needs.

Convincing people to make changes in their diet is not a simple task. The emphasis placed on the need to change the dietary preferences in large populations and the reduction in the amount of meat consumed, however, cannot be based solely on rational arguments presented to the general public by doctors or scientists. There are many psychological mechanisms which make it difficult to implement even small diet changes despite the vast empirical evidence and medical recommendations [110,111]. This is probably most clearly demonstrated by the fact that people who suffer from health conditions such as diabetes or coronary disease are often unwilling to introduce such changes to their diet that may even help them fully manage their conditions, and sometimes considerably prolong their lives.

One of the solutions may be the use of new technologies and media [112]. Directly reaching the recipient, conducting monitoring, and providing feedback (though not until recently was unrealistic) in the era of advanced technology and a wide network of social media, is possible. It may also be necessary to develop personalized programs for different segments of consumers.

Recommended changes in the diet should be carried out taking into account the fear of food novelty. This applies not only to the form or presentation of products, but mainly to cultural proximity and the level of food processing. The easier to identify the product, and the closer it is to familiar foods, the easier it will be to accept and the greater the likelihood of inclusion in the diet.

Although the contemporary human is considered mainly as an outcome of psycho-social processes, the evolutionary roots of humanity should not be forgotten. These may involve a desire for meat. Without properly addressing these deep psychological needs, we are not going to shape a more sustainable profile of human consumption and nutrition. Therefore, research involving human behavior should become a more integral part of nutrition science.
